# Judges versus artificial intelligence in juror decision-making in criminal trials: Evidence from two pre-registered experiments

**DOI:** 10.1371/journal.pone.0318486

**Published:** 2025-01-30

**Authors:** Eiichiro Watamura, Yichen Liu, Tomohiro Ioku

**Affiliations:** 1 Graduate School of Human Sciences, Osaka University, Suita, Osaka, Japan; 2 Center for International Education and Exchange, Osaka University, Suita, Osaka, Japan; Uniwersytet Jagiellonski w Krakowie, POLAND

## Abstract

**Background:**

Artificial intelligence (AI) is anticipated to play a significant role in criminal trials involving citizen jurors. Prior studies have suggested that AI is not widely preferred in ethical decision-making contexts, but little research has compared jurors’ reliance on judgments by human judges versus AI in such settings.

**Objectives:**

This study examined whether jurors are more likely to defer to judgments by human judges or AI, especially in cases involving mitigating circumstances in which human-like reasoning may be valued.

**Methods:**

Two pre-registered online experiments were conducted with Japanese participants (Experiment 1: N = 1,735, Mage = 48.4; Experiment 2: N = 1,731, Mage = 48.5). Participants reviewed two murder trial vignettes and made sentencing decisions (1 = suspended sentence; 8 = prison sentence) under two conditions: trials with and without mitigating circumstances.

**Results and conclusion:**

Across both experiments, participants showed no preference for deferring to human judges’ or AI judgments when making sentencing decisions. While suspended sentences were more common in cases with mitigating circumstances, this tendency was unrelated to the judgment source. These findings suggest that jurors do not inherently avoid algorithmic judgments and may consider AI opinions on par with those of human judges in certain contexts. However, whether this leads to improved decision-making quality remains an open question, as objectivity (a strength of AI) and emotional considerations (a safeguard for fairness) may interact in complex ways during juror deliberations. Future research should further explore how these factors influence juror attitudes and decisions in diverse trial scenarios, taking into account potential biases in existing literature.

## Introduction

The use of artificial intelligence (AI) in courts is accelerating [1, 2]. AI can refine information extracted from testimony and text [3–5], analyze surveillance camera images to identify perpetrators [6], classify investigative materials, and prepare trial transcripts efficiently [7, 8]. It has also proven useful as an assistant to judges, for example, by determining which evidence and testimony are conclusive and reliable to prove facts [9], identifying similar cases, and suggesting sentences based on precedents [10]. Expectations are growing for the realization of robot judges (also known as “AI judges” and “algorithmic judges”), which can replace human judges and make decisions automatically based on vast amounts of case data [11–14].

These benefits are substantial; however, they must be balanced against the unique challenges posed by AI in judicial contexts. Using AI in the judicial domain presents challenges that must be resolved. As AI learns from training data, it may incorporate biases contained in the data [15]. Any discrimination based on race, gender, social background, or other demographic factors created by bias in training data would threaten the fairness of judgments. The supposed “black-box problem” is also a major concern [16, 17]. Although accountability is an essential element for decision-making in court [e.g., 16–20], the process of how the AI arrives at a particular conclusion or judgment is opaque because of the lack of access to the internal workings of the algorithm, the decision criteria, and the learning process [21, 22]. This opacity makes it difficult for AI to meet the accountability criterion at present, although some argue that the opacity issue is not important, as the human mind is similar (e.g., [23]).

Nevertheless, AI offers significant potential to address human limitations, particularly in eliminating cognitive biases and emotional influences that often affect human judgment. These advantages suggest that its use in courtrooms is not only inevitable but also essential for achieving fairer and more efficient trials. People are likely to make judgments based on accessible memories, such as recent or memorable cases (availability heuristic; [24]), or make quantitative judgments according to numerical information given in advance, such as the prosecutor’s plea or the amount of damage claimed (anchoring; [25]). Moreover, people often do not consistently judge the same case (noise; [26]). Emotions, such as anger and sadness, can influence judgments, which can change decisions [27–29]. Of course, issues such as dealing with bias in AI training data and ensuring transparency in the decision-making process must be overcome, but by mitigating cognitive bias and emotional influence, AI has the potential to greatly improve the fairness and consistency of judicial decisions. Furthermore, organizing large volumes of documents and evidence using AI would significantly shorten the time required to reach a judgment and reduce litigation delays [30, 31]. AI can also reduce the labor costs and time required to run a courtroom, as automation, especially of simple and repetitive tasks, will make courts more cost-efficient [32, 33]. In addition, AI-powered online platforms and chatbots will make it easier for the public to obtain legal advice and assistance [34, 35], improving access to legal services [36]. Owing to the many potential benefits and those described above, the possibility of AI being introduced into the courts is now realistic [37]. Thus, our focus should not be on whether AI should be introduced into the courtroom but rather on the emerging question of how to use it in courtroom settings successfully.

AI could be used in criminal trials with citizen participation, such as jury and adversarial trials [38]. In these courts, there could be a procedure whereby juries make their decisions based on the judge’s and AI’s verdicts. However, it is largely unclear whether jurors are more likely to defer to a judge or an AI, particularly if their decisions are inconsistent. If jurors place too much trust in the AI’s judgment or ignore helpful information from the AI and blindly follow the judge’s opinion, they could come to a biased decision. The jury’s job is to determine the facts and render a fair verdict. If jurors rely too much on the AI or the judge, there is a risk that they will become mere bystanders and lose their ability to make independent judgments. Consequently, for maintaining the fairness and credibility of future justice, it is essential that jurors understand the differences between the opinions of AI and judges and that the degree to which they value one over the other is examined. In particular, we must be prepared for extreme situations in which jurors give more weight to either a judge or an AI when the two are in conflict. Identifying the conditions under which a judge or AI is more likely to be deferred to can clarify the division of roles between the two in a human–machine hybrid system [39] and facilitate the optimization of the system so that one side’s opinion is not neglected. This study is a forerunner to this approach.

### Literature review

Do jurors find the judgments of human judges or AI more helpful? Some studies show that one is more trusted than the other, whereas others conclude that there is no difference. These conflicting outcomes have led to a complex and confusing debate [40] regarding trust in AI and human judgment. An experiment conducted with 958 Dutch people found that an automated AI decision-making process was rated as more useful than human experts in important judicial decisions, such as proceedings to initiate a lawsuit [41]. In situations where objective fairness is important, AI is more likely to be considered superior to human experts [42–44]. Despite this high regard for the fairness of AI, several studies have also demonstrated an algorithm avoidance tendency, indicating that people do not consider its decisions as acceptable as those of human experts [45, 46].

However, existing research has primarily focused on civil cases or scenarios in which AI and human judgments do not directly conflict, leaving a gap in understanding how jurors navigate situations wherein AI and human judges provide inconsistent recommendations. In Chen et al.’s [37] experiment, participants rated a human judge’s decision as being procedurally fairer than the decision made by a robot judge across three trials (consumer retail arbitration, bail decision, and sentencing decision). Meanwhile, Hayashi et al.’s [47] experiment examined the sentencing decisions of citizens acting as jurors in a trial against a defendant in a robbery-homicide case. The results showed that participants deferred to both expert and AI sentencing requests but deferred more to the opinions of human experts when they wanted a heavier sentence in more crucial decisions. In an experiment by Yalcin et al. [48], participants read a fictitious vignette about going to a local court to divorce their partner and were asked about their trust in an algorithmic judge or human judge and their intention to file a lawsuit. The results showed that participants placed more trust in the human judge and expressed their intention to file the case in a court where a human judge would decide, rather than one where an algorithmic judge would rule.

The reason for avoiding AI’s judgments is believed to be that it lacks the human-like ability to consider the subjective factors behind the evidence and law: that is, emotional, moral, and social factors [49, 50]. Indeed, in Yalcin et al.’s [48] divorce litigation experiment, AI was less trusted than in other cases when the partner who was about to leave was experiencing mental health problems. Conversely, there is evidence that if an AI has human-like capabilities, the tendency toward algorithm avoidance disappears. For instance, in an experiment by Watamura et al. [17] using trial clips, a robot judge who was empathetic toward the defendant was trusted by the participants as much as a human judge. The robot’s sentencing decision was also accepted to the same extent as the judge’s. These results suggest that AI is avoided because its decision-making is mostly not human-like.

Despite these insights, there is a lack of research examining juror behavior in criminal trials in which AI and human judges present conflicting judgments, particularly in the presence of mitigating circumstances that require nuanced, human-like understanding. To summarize the results of previous studies, AI decisions are likely to be used as frequently as those of judges when human feelings do not need to be considered. However, when such sentiments are expected to be taken into account, a tendency to avoid algorithms is likely to emerge. In the context of procedural justice, studies have shown that the decisions of authority figures believed to understand the feelings of the parties involved are more likely to be accepted [51, 52]. Procedural justice refers to the perceived fairness of the processes that lead to outcomes, emphasizing transparency, impartiality, and the ability to voice concerns [53]. As AI is regarded as lacking the ability to understand emotions to the extent humans do [49, 50], it is unlikely to be trusted in court cases where this ability is required. In a criminal trial with citizen participation, where there are mitigating circumstances requiring human-like competence, the jury will defer to the judge’s decision rather than a decision made by the AI. In trials without mitigating circumstances, jurors are likely to defer equally to the judge’s and the AI’s decisions.

### The present study

This study conducted two pre-registered online experiments to examine whether jurors were more likely to defer to the human judge or AI’s judgment when making decisions in criminal trials. In the context of Japan’s lay judge system, professional judges and lay judges collaborate to deliberate and decide both the verdict and sentencing. This study assumes such a system, wherein jurors are presented with both a judge’s and an AI’s opinions to aid their decision-making. This differs from Anglo–American jury trials, which generally separate jurors’ verdicts from judges’ sentencing responsibilities. Mock jury participants read case vignettes and decided whether the defendant should be given prison or a suspended sentence. They were presented with decisions recommended by a judge and AI to examine which decision participants chose when these decisions conflicted, one recommending a prison sentence and the other a suspended sentence. The outcome measure was the participants’ sentencing decision, ranging from 1 (suspended sentence) to 8 (prison sentence). If more weight were given to the judge’s decision, the participants would choose a prison sentence under the conditions in which the judge imposed a prison sentence and a suspended sentence under the conditions in which the judge imposed a suspended sentence. No such difference would be seen if the AI were mentioned as much as the judge. Thus, the following hypothesis was tested:

Hypothesis: A judge’s judgment is more likely to be deferred to than that of AI in cases with mitigating circumstances.

To establish mitigating circumstances, Experiment 1 used a case in which a defendant who was a victim of domestic violence murdered her husband, and Experiment 2 used a case in which a defendant murdered his mother, who had terminal cancer. These vignettes were selected to reflect criminal cases frequently reported in Japanese media, such as domestic violence and familial homicide, which are both socially and legally significant issues in Japan. By using scenarios that participants might find relatable and relevant, we aimed to enhance the ecological validity of the study and ensure that the cases would engage participants’ judgments authentically. This selection aligned with the study’s goal of investigating how mitigating circumstances and decision-making sources (AI vs. human judges) influence jurors’ decisions.

This study’s contribution to the literature lies in examining jurors’ preferences for either judges or AI when making decisions by directly manipulating an independent variable that is not good for AI: in this case, the presence or absence of mitigating circumstances. Although Yalcin et al.’s [48] experiment had a similar manipulation, it was conducted in a civil case trial. Thus, to our knowledge, this study is the first to take such an experimental approach in a criminal case.

## Experiment 1

Participants were randomly assigned to one of four groups that combined two conditions of mitigating circumstances (present vs. absent) and two conditions of the decision pattern (judge vs. AI recommending a prison or suspended sentence). The decision patterns were designed to always conflict, ensuring that participants had to decide whether the defendant should receive a prison sentence or a suspended sentence. This setup allowed us to directly test participants’ deference to the judge or AI under different conditions.

### Methods

This online experiment, as well as Experiment 2, was approved by the Ethical Review Committee of the Department of Behavioral Sciences, Graduate School of Human Sciences, Osaka University (Approval number: HB023-125), and was conducted based on pre-registered procedures and analysis methods. As described in the subsection on procedures below, informed consent was obtained from participants in electronic form. The pre-registration details and raw data are available on the Open Science Framework.

#### Participants

The target sample size (N = 1,200) was based on Yalcin et al. [48], who used a similar 2 × 2 experimental design to detect small to medium effect sizes (f = 0.10) with 80% power at α = 0.05. To account for potential exclusions due to attention checks, we oversampled by 500 participants. Participants were randomly selected from a Japanese Internet research firm’s panel of individuals aged 18 years or older. Invitations were mailed to 20,884 participants to ensure sufficient response rates, accounting for an estimated 30% exclusion rate from attention checks (see the next subsection). Recruitment for this study began on December 11, 2023, and ended on December 14, 2023. The panel was demographically unbiased, drawn from all over Japan, and largely representative of the Japanese population. As 98.5% of the Japanese public is ethnically Japanese [54], the authors decided not to collect ethnicity data from the survey participants. Participants were given a reward through shopping points redeemable for Amazon gift cards and other products.

#### Procedures

The procedure followed in this study was based on those of previous studies that used vignettes [47, 48]. Of the panel to whom invitations were sent, those participants who read the instructions and provided informed consent by clicking the “I participate” button were directed to the experimental screen. A total of 1,735 participants—slightly more than the target number—completed the questionnaire (869 men and 866 women, *M*_age_ = 48.4, *SD* = 14.9). First, participants were informed about the task: “As a juror in a criminal trial, decide whether the defendant should be sentenced to prison or given a suspended sentence.” Next, they read an introduction to the judge and the AI:

In this jury trial, both the judge and the AI encourage you to make decisions against the defendant. The judge is a very experienced veteran, and the AI has machine-learned a great deal of case law. Detailed information about the defendant’s background, criminal record information, and mitigating circumstances are distributed equally to the judge and the AI.

Next, the participants read a case vignette (see S1 Appendix). The trial vignette, which was a condition with mitigating circumstances, described a female defendant accused of murdering her husband; the woman had been regularly subjected to domestic violence by the victim. Out of a desire to escape the pain, she strangled the victim in his sleep using a rope. The trial vignette for the without-mitigating-circumstances condition described a female defendant who was fed up with her husband, who suffered from a chronic illness, and strangled him in his sleep.

For each condition, half of the participants were asked to read the following description: “The judge ruled that the defendant should be sentenced to prison, and the AI ruled that the defendant should be given a suspended sentence.” The other half were asked to read this description: “The judge ruled that the defendant should be given a suspended sentence, and the AI ruled that the defendant should be sentenced to prison.” Participants answered the following questions in order:

(1) “If you were present at this trial as a juror, would you decide that the defendant should be given a prison sentence or a suspended sentence?” Participants were asked to answer this question on an 8-point scale (1 = suspended sentence to 8 = actual sentence).

(2) As a manipulation check, the participants were asked to respond to three items related to the mitigating circumstances (“I think there are circumstances in favor of the defendant,” “It is too much to put all responsibility on the defendant,” and “The defendant is not completely at fault”) on a 6-point scale ranging from 1 (“I do not agree at all”) to 6 (“I strongly agree,”).

(3) Finally, as an attention check to ensure that they had read the vignette properly, the participants were asked to answer “Yes” or “No” to three items describing the details of the vignette (e.g., “The female defendant killed her husband using a knife”). These items were not intended to assess participants’ memory but rather to verify their engagement with the experimental materials. This approach was taken to maintain the validity of the responses, as online experiments may include participants who answer without fully engaging with the task.

### Results

The analysis was performed as per the pre-registration. HAD version 17.3 [55] was used for analysis. First, 948 participants who incorrectly answered at least one of the three attention check questions were excluded (see S3 Appendix for results of the following analyses with a full sample). A total of 787 participants (424 women, 363 men, *M*_age_ = 49.91, *SD* = 14.68) from the four groups were included in the analysis, ranging from 168 to 233 in each group. A post hoc power analysis using G*Power confirmed that with a small effect size (f = 0.10), α = 0.05, and a sample size of 787, this study achieved a power of 0.80. This ensures the sample size was sufficient to detect small effects reliably. Analyses were conducted to confirm the validity of the experimental manipulation of the mitigating circumstances. The reliability coefficients for the three items were sufficiently high (α = .86); thus, a t-test was conducted using averaged scores. The results showed that the two groups exposed to the with-mitigating-circumstances condition (*M* = 4.48, *SD* = 0.95) considered that there were significantly greater mitigating circumstances than the two groups in the without-mitigating-circumstances condition (*M* = 3.42, *SD* = 1.08) (*t*(785) = -14.14, *p* < .00, Hodge’s g = -1.02, 95% CI = -1.17, -0.87).

**Fig 1:**
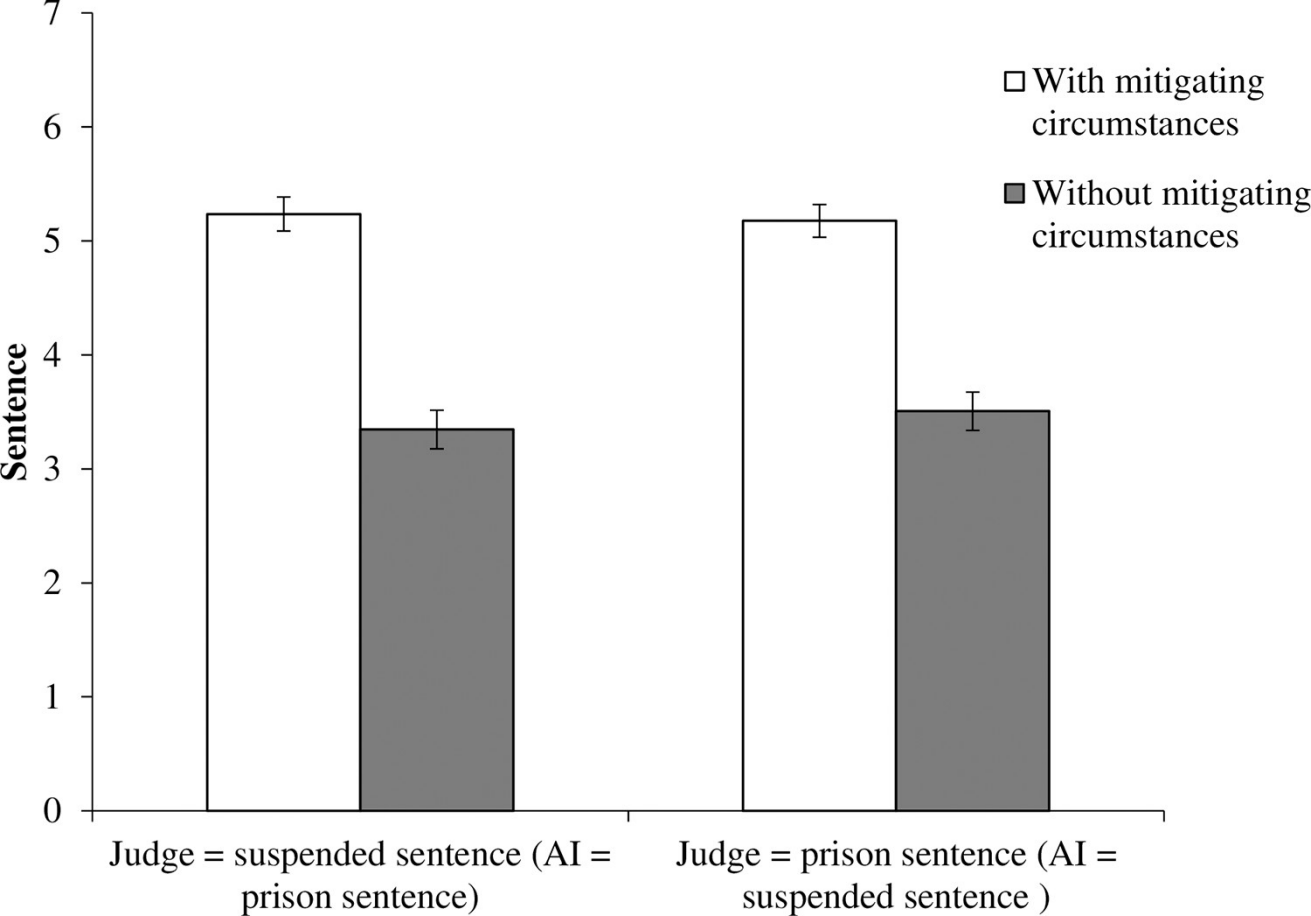
Jury judgments against defendants in Experiment 1. Error bars indicate standard errors.

As the experimental manipulation of the mitigating circumstances was valid, an analysis of variance was conducted with the participants’ judgments measured on an eight-point scale as the dependent variable, and mitigating circumstances and the decision patterns of the judge and AI as the two independent variables. No interaction was found (*F*(1,783) = .49, *p* = .49, partial η2 = .00) (Fig 1). There was a significant main effect of the mitigating circumstances, with prison sentences being more likely to be chosen by the two groups in the without-mitigating-circumstances condition (*M* = 5.20, *SD* = 2.09) than in the two groups in the with-mitigating-circumstances condition (*M* = 3.43, *SD* = 2.32) (*F*(1,783) = 127.11, *p* < .00, Partial η2 = .14). No main effect of judge and AI decision patterns was confirmed (*F*(1,783) = 0.10, *p* = .75, partial η2 = .00).

### Discussion

Our hypothesis was not supported because the participants’ judgments in the with- and without-mitigating-circumstances conditions did not change according to the decision patterns of the judges and AI. We expected the participants to be more likely to defer to the judge than the AI in the with-mitigating circumstances condition. Specifically, we expected that participants’ decisions in a case in which the defendant, who had suffered from domestic violence, had murdered her husband would be influenced by the judge’s decision and that they would be more likely to opt for a prison sentence if the judge favored a prison sentence and for a suspended sentence if the judge favored a suspended sentence. We predicted that such an advantage would not be found in the absence of mitigating circumstances. The experimental results showed that suspended sentences were more likely to be chosen in the condition with mitigating circumstances than in the condition without mitigating circumstances (i.e., main effect). Notably, there was no main effect of the judges’ decision patterns of accepting AI decisions in this study. Previous studies have shown that judges’ decisions are more accepted than AI’s [37, 48]. This algorithm avoidance tendency [45, 46] might have not been confirmed in the present study due to the particular cases used in the experimental material. For example, participants might have thought that the relationships between the couples were too complex to be understood even by a human (judge). As a result of the judge being less likely to be deferred to, the difference in the ease of deference of the AI might have been reduced, and the algorithm avoidance tendency may not have been confirmed. To examine the generalizability of the findings, Experiment 2 employed a scenario of murder due to caregiver burnout instead of the mariticide scenario used in Experiment 1. Caregiver murder reflects the diversity of morally salient contexts that could influence juror decision-making [56]. Differences in public perceptions of these crimes may offer additional insights into how algorithm avoidance or reliance manifests in varying emotional and moral contexts.

## Experiment 2

In the with-mitigating-circumstances condition of Experiment 2, we used a case in which a male defendant murdered his terminally ill mother. As in Experiment 1, four groups were established, which were exposed to a combination of the mitigating circumstance conditions (with vs. without) and the decision patterns of the judge and AI (the judge sentenced the defendant to prison, and AI gave the defendant a suspended sentence vs. vice versa). Participants were randomly assigned to one of the groups.

### Methods

#### Participants

Our aim was to recruit a sample of 1,700 people. Invitation e-mails were sent to 21,188 randomly selected Japanese panelists aged 18 years or older who were registered with the same Internet research company as in Experiment 1. Recruitment for this study began on December 18, 2023, and ended on December 21, 2023. Participants from Experiment 1 were excluded from this study.

#### Procedures

The 1,731 participants (867 men and 864 women, *M*_age_ = 48.5, *SD* = 14.9) who provided informed consent were directed to an experimental screen and instructed to decide whether they would serve the defendant with a prison or a suspended sentence. After reading an introduction stating that the judge and AI were given the same information, they were presented with a trial vignette (see S2 Appendix). The vignette with-mitigating-circumstances condition illustrated a case in which an unemployed male defendant had murdered his elderly mother. The defendant had no choice but to kill his mother because she wanted to die when her terminal cancer worsened. In the trial vignette without mitigating circumstances, a male defendant killed his mother because he was unhappy with being pressured to leave the house. For each condition, half of the participants were told that the judge recommended a prison sentence, and the AI suggested a suspended sentence, whereas the other half were told that the judge wanted a suspended sentence, and the AI proposed a prison sentence. Participants were then asked to rate (1) their judgment against the defendant on an 8-point scale (1 = suspended sentence to 8 = prison sentence), (2) three manipulation check items of mitigating circumstances on a 6-point scale ranging from 1 (“I do not agree at all”) to 6 (“I strongly agree,”), and (3) three attention check items (e.g., “The offenders did not have regular jobs at the time of the incident”), to which they answered “Yes” or “No.”

### Results

After excluding 871 individuals who answered one or more questions incorrectly during the attention check (see S4 Appendix for results of the following analyses with a full sample), 860 individuals (446 women, 414 men, *M*_age_ = 50.62, *SD* = 14.53) remained for analysis (194–238 in each group). A post hoc power analysis using G*Power confirmed that with a small effect size (f = 0.10), α = 0.05, and a sample size of 860, our study achieved a power of 0.83. As the reliability coefficients for the three items on mitigating circumstances were sufficiently high (α = .85), t-tests were conducted using averaged scores. The results showed that the two groups in the with-mitigating-circumstances condition (*M* = 4.11, *SD* = 0.94) considered that there were significantly greater mitigating circumstances than did the two groups in the without-mitigating-circumstances condition (*M* = 2.87, *SD* = 1.09), *t*(858) = -17.58, *p* < .00, Hodge’s g = -1.20, 95% CI = -1.34, -1.06), confirming that we manipulated the mitigating circumstances as intended.

**Fig 2:**
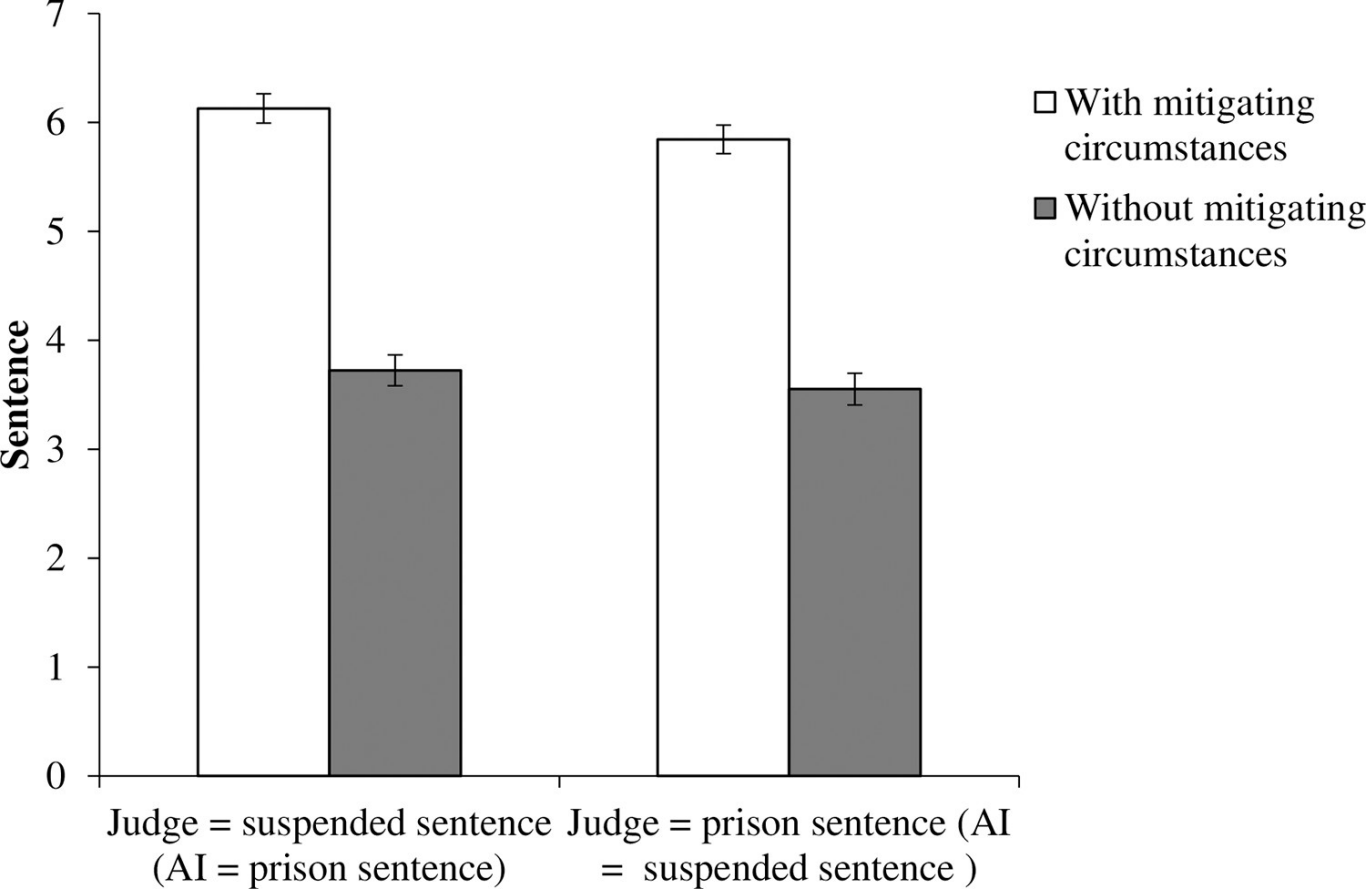
Jury judgments against defendants in Experiment 2. Error bars indicate standard errors.

An analysis of variance was conducted with the sentencing decision measured on an 8-point scale as the dependent variable and the two independent variables of the mitigating circumstances and decision patterns of the judge and AI. The results replicated those of Experiment 1. An interaction effect was again not found (*F*(1,856) = .16, *p* = .69, partial η2 = .00) (Fig 2). A significant main effect of the circumstances was confirmed in Experiment 2, with the two groups in the without-mitigating-circumstances conditions (*M* = 5.98, *SD* = 1.87) being more likely to choose a prison sentence than the two groups in the with-mitigating-circumstances condition (*M* = 3.64, *SD* = 2.18) (*F*(1,856) = 288.79, *p* < .00, partial η2 = .25). A main effect of judge and AI decision patterns was, again, not confirmed (*F*(1,856) = 2.73, *p* = .10, partial η2 = .00).

### Discussion

Although we used a different experimental vignette in Experiment 2 to that in Experiment 1, our hypothesis was not still supported. We found no interaction between the presence of mitigating circumstances and the judge’s or AI’s decision patterns; therefore, our prediction that judges’ decisions would more likely be deferred to in the condition with mitigating circumstances was again not confirmed by the results of Experiment 2. Importantly, unlike previous studies [37, 48], Experiment 2 showed no evidence that judges were more likely to be deferred to than AI: Regardless of whether the judge or the AI recommended a prison sentence, the participants’ judgments did not change, and their judgments were only influenced by the presence or absence of mitigating circumstances. The lack of interaction between AI and judge decisions, which was also confirmed in Experiment 2 as well as Experiment 1, may be because the participants were equally distant from the factors inherent in both judges. This suggests that the mitigating circumstances themselves played a more central role in influencing participants’ judgments than the perceived humanistic competence or objectivity of the decision-makers. In other words, our results emphasized the possibility that participants prioritized the moral weight of the mitigating circumstances over the decision-making source, whether human or AI.

## General discussion

Are jurors more likely to defer to the judgments of human judges or those of AI? In the future, AI will likely be used in citizen-participatory criminal trials, such as jury and adversarial trials [38, 57]. The future fairness and credibility of justice depends on jurors understanding the differences between the opinions of AI and those of judges and the degree of weight they give to each. Additionally, cultural influences might have shaped jurors’ decision-making. In societies in which judicial systems emphasize impartiality and procedural fairness, jurors may place equal trust in AI and human judges, reducing the impact of algorithm avoidance tendencies observed in other studies. By examining the conditions under which each type of judge is likely to be used, we can create a system that can evaluate human judges and AI judges in a human–machine hybrid system without bias [39].

We examined participants’ reactions in situations in which the judgments of a human judge and an AI’s judgments conflicted. We expected that participants would be influenced by the decision of the one they preferred to defer to more. The presence or absence of mitigating circumstances was the key factor and independent variable. As AI is deemed to lack the human-like ability to consider emotional, moral, and social factors [49], a judge’s judgment should be more likely to be deferred to than that of an AI in a trial with mitigating circumstances. Thus, we predicted that jurors would be more likely to choose a prison or suspended sentence if the human judge supported it. However, the results of the two experiments showed that mitigating circumstances were not related to the likelihood of participants deferring to either the judge’s decision or that of the AI. Instead, participants focused exclusively on the presence or absence of mitigating circumstances (i.e., main effect): A female victim of domestic violence who murdered her husband (Experiment 1) and a male defendant who murdered his mother, who had terminal cancer and wanted to die (Experiment 2), were more likely to be granted suspended sentences than prison sentences. Thus, although the presence or absence of mitigating circumstances influenced participants’ judgments, we found no evidence to predict that the presence or absence of mitigating circumstances made a difference in the likelihood that a human judge or an AI was used. In summary, jurors’ tendency to favor more lenient sentences in the face of mitigating circumstances remained unaffected, regardless of who suggested which sentences. These findings contribute to the literature in two ways. First, they showed that jurors did not favor the judge or the AI in making decisions in criminal cases. Second, they identified juror performance in situations where the judge and AI were in conflict. The lack of support for our hypothesis may reflect fundamental differences in how AI and human judges approach complex judicial scenarios. While human judges may rely on moral reasoning and emotional intuition, AI’s decision-making is grounded in algorithmic logic, which might not align with jurors’ expectations in cases involving nuanced emotional or social contexts.

### Theoretical implications

Notably, this study did not confirm algorithm avoidance tendencies [45, 46], even though the literature has repeatedly shown that AI is not particularly preferred in ethical decision-making situations [58, 59]. Indeed, in Yalcin et al.’s [48] experiment, human judges were preferred in divorce cases with emotional complexity (compared with other cases). However, neither of our experiments confirmed an algorithm avoidance tendency. Unlike Yalcin et al. [48], who used civil case vignettes, this study used criminal case vignettes. In criminal trials, other factors may offset the effects of algorithmic avoidance. For example, objectivity is likely to be highly valued in criminal trials [60, 61]. Therefore, even if tendencies to avoid algorithms negatively affected the participants’ responses in the study, the positive impact of expectations of objectivity could have been offset, which is one of the strengths of AI. As a result, the likelihood of deferring to the AI might have emerged at the same level as the likelihood of deferring to the human judge. Nevertheless, the fact that some studies have demonstrated algorithm avoidance in criminal trials [37, 47] suggests that this explanation may be insufficient. Rather, the results may be due to the unique setting of our study, in which the judgments of the human judge and those of the AI conflicted. In Yalcin et al.’s [48] experiment, participants were assigned to either the AI or judge condition (i.e., between-participant design), which might have confirmed the algorithm avoidance tendency. Alternatively, familiarity with recent technologies, such as ChatGPT, might have reduced negative attitudes toward AI. Another possibility is that there is potentially a large amount of negative data, such as those generated in our study, which have not been reported (i.e., publication bias). Studies that show a tendency to avoid algorithms may be more likely to be reported because people are cautious about AI. Therefore, if more negative data are reported, this would further illustrate people’s current attitudes towards AI.

### Practical implications

An important implication of this study is the possibility that jurors would not be biased toward one decision or another if a human–machine hybrid system [39] were introduced in jury trials. The participants were aware of the existence of mitigating circumstances. Nevertheless, our findings suggest that both human judges’ and AI’s judgments are deferred to equally in criminal trials, even in cases in which humanistic competence to understand mitigating circumstances is particularly necessary. This balance highlights the potential for hybrid systems to reduce undue reliance on either human judges’ emotional intuitions or AI’s algorithmic objectivity, offering a complementary dynamic in juror deliberations [62]. However, AI’s limitations in understanding subjective factors, such as empathy and morality, cannot be overlooked. While AI excels in objectivity and consistency, it lacks the capacity to perceive and evaluate emotional and moral nuances, which are often critical in judicial contexts. For instance, jurors may expect judges to interpret mitigating circumstances with empathy and moral reasoning—abilities that AI currently cannot replicate. This underscores a crucial gap in AI’s applicability, particularly in cases that demand a nuanced understanding of human emotions and social complexities. Previous studies have shown that mitigating circumstances reduce perceptions of culpability and influence jurors to recommend more lenient sentences [63, 64]. However, these studies generally did not consider the added influence of a judge’s recommendation. Theoretical frameworks, such as the dual-process theory of decision-making [65], suggest that jurors might rely more heavily on a judge’s recommendation in emotionally charged cases involving mitigating circumstances. This is because judges, as legal experts, may be perceived as better equipped to balance emotional and rational considerations, particularly when mitigating factors complicate the moral evaluation of the case. We confirmed that jurors valued humaneness, even in AI-supported trials. In cases with mitigating circumstances, people reduce their evaluations of a defendant’s culpability or justify a lighter penalty [66, 67]. In our two experiments, the manipulation of mitigating circumstances strongly affected the participants’ judgments.

### Limitations and future research

This study has several limitations. First, it remains unclear why there were no differences between participants’ deference to AI and that to human judges. Jurors might have viewed AI as being equally capable of considering mitigating circumstances as human judges, or as noted earlier, another positive benefit of deferring to AI might have been expected. People regard the criteria for moral judgment as being different between AI and human judgments [68]; thus, although the participants deferred to them to the same degree, it was not necessarily for the same reason. Second, the participants in the online experiment were inauthentic. We included a three-question attention check, because online respondents tend to answer questions without reading them [69]. Some participants might have engaged superficially, likely motivated by the financial incentive, which contrasts with those who approached the task seriously, providing thoughtful and authentic responses. We decided to exclude participants who failed at least one attention check to maintain the reliability of our dataset. While this approach ensures the inclusion of engaged participants, it might have inadvertently excluded individuals who genuinely understood the manipulations but overlooked specific vignette details. Future research could explore alternative methods for verifying engagement to better balance data quality with participant inclusion. This should be verified in another setting in which participants answer seriously, such as in a mock guessing experiment. Future research could also investigate the general public’s understanding of how AI is trained and operates, as well as the impact of these beliefs on trust and decision-making. This would help clarify whether algorithm avoidance is sometimes justified and how perceptions of AI’s capabilities influence behavior. Furthermore, the specific emotional and ethical context of the cases presented in our experiments might have overridden the expected influence of the perceived expertise or empathy of judges and AI. Future research should further explore how contextual factors shape jurors’ reliance on different decision-making agents. Another possibility is that the increasing integration of AI technologies in daily life has reduced participants’ skepticism toward AI, leading them to treat its recommendations as comparable to those of human judges. Future research should examine whether familiarity with AI or shifts in societal attitudes influence its perceived comparability to human judges.

## Conclusion

Are jurors more likely to defer to the judgments of human judges or AI? We have conducted two experiments to prepare for the introduction of AI in criminal trials with citizen participation. In trials with mitigating circumstances, we predicted that participants would base their verdicts on the recommended decision of a human judge. The results of the experiments showed that the presence or absence of mitigating circumstances affected participants’ judgments. However, no evidence supported the prediction that the presence or absence of mitigating circumstances would make a difference in the extent to which they deferred to a human judge or to AI. Consequently, unlike previous studies, we found no evidence of algorithm avoidance. In the context of the lack of known algorithm avoidance in jury trials, our study, using mitigating circumstances as a threshold, raised the possibility that we did not bias jurors toward the decisions of the human judge or the AI. The negative data obtained in this study, where there was no difference in the extent of deference to the judge and AI, suggest that juror decision-making may improve if both are given equal consideration in trials following the introduction of AI. Considering the results of this study and the various benefits that AI brings, we could be more positive about the introduction of AI in criminal courts. However, as this study was an online survey, future research should investigate the ease of mentioning judges and AI in more realistic mock jury experiments and report whether avoidance of algorithms is observed. Furthermore, the findings of this study carry broader societal and ethical implications. As the use of AI in criminal justice systems becomes more prevalent, it is critical to address such issues as the transparency, accountability, and fairness of AI algorithms. Policymakers should consider the potential risks of algorithmic bias and the need for stringent oversight mechanisms to ensure ethical AI integration in legal settings. Additionally, public education campaigns could be designed to improve understanding and trust in AI-assisted decision-making, thereby reducing skepticism and fostering informed participation in hybrid systems. Future research should explore the long-term impacts of AI on public perceptions of justice and develop frameworks to balance technological advancements with societal values.

## Supporting information

S1 Appendix Experiment 1 vignette. (DOCX)

S2 Appendix Experiment 2 vignette. (DOCX)

S3 Appendix Jury judgments in Experiment 1 (full sample analysis). Error bars indicate standard errors.
(DOCX)

S4 Appendix Jury judgments in Experiment 2 (full sample analysis). Error bars indicate standard errors.
(DOCX)
