# Supplementary material for: Judges versus artificial intelligence in juror decision-making in criminal trials: Evidence from two pre-registered experiments
Source: PLoS One. 2025 Jan 30;20(1):e0318486. doi: 10.1371/journal.pone.0318486 (PMC11781698; doi:10.1371/journal.pone.0318486)
Supplement: S1 Appendix — (DOCX) [file pone.0318486.s001.docx]

**Supporting information**

**S1 Appendix Experiment 1 Vignette.**

Case in which the defendant suffered from domestic violence and murdered her husband

(With-mitigating-circumstances condition)

A jury trial involving a 48-year-old woman who murdered her 54-year-old husband while he was sleeping was held at the Tokyo District Court. According to the prosecution’s argument, at approximately 1:00 a.m. on March 10, 2022, the woman allegedly strangled her husband, who was a habitual drinker, to death with a rope while he was asleep and left his body. The husband had a history of nightly alcohol consumption and had subjected the woman to domestic violence; she was observed running barefoot and crying in the cold of February with her face covered in blood. Although the husband had a good reputation among their neighbors, he had been previously arrested for assault in 2004 and 2013. On the day of the incident, after confirming that her husband was snoring and fast asleep, the woman tied the end of a rope to a post and wrapped it around his neck, saying, “It's now or never.” During this jury trial, an AI-based sentencing support system was utilized.

(Without-mitigating-circumstances condition)

A jury trial involving a 48-year-old woman who murdered her 54-year-old husband while he was sleeping was held at the Tokyo District Court. According to the prosecution’s argument, at approximately 1:00 a.m. on March 10, 2022, the woman allegedly strangled her husband, who was a habitual drinker, to death with a rope while he was asleep and left his body. Despite her husband having a good reputation among their neighbors, his chronic illness had worsened, rendering him unable to work for many days, and he often complained to the woman. Worried about her future, the woman took her husband to the hospital and often sought advice from her friends. Troubled by the lack of improvement in her husband's chronic illness over time, the woman began to feel she had no choice but to let him die. On the day of the incident, after confirming that her husband was snoring and fast asleep, the woman tied the end of a rope to a post and wrapped it around his neck, saying, “It's now or never.” During this jury trial, an AI-based sentencing support system was utilized.
