# Supplementary material for: Judges versus artificial intelligence in juror decision-making in criminal trials: Evidence from two pre-registered experiments
Source: PLoS One. 2025 Jan 30;20(1):e0318486. doi: 10.1371/journal.pone.0318486 (PMC11781698; doi:10.1371/journal.pone.0318486)
Supplement: S2 Appendix — (DOCX) [file pone.0318486.s002.docx]

**Supporting information**

**S2 Appendix Experiment 2 Vignette.**

Case in which the accused murdered his mother, who had terminal cancer.

(With-mitigating-circumstances condition)

A jury trial involving a 52-year-old man who murdered his 82-year-old mother who was suffering from terminal cancer was held at the Osaka District Court. According to the prosecution, the man was suspected of strangling his mother with a rope and leaving her body on the banks of the Yodo River around 23:00 on May 22, 2022. Four years before the incident, the man quit his job to care for his mother, who was battling cancer. As her condition deteriorated, the mother expressed a desire to die, stating, “I want to die because there is no hope for me to get better.” The man encouraged her, saying, “Don’t say that.” However, he felt that he would fall ill with her if he did not do something about it, so he decided to kill his mother out of necessity. In the evening of the incident, he took his mother out for a walk and headed to the crime scene by train. Seeing that no one was around, he took out a rope from his bag, strangled her from behind, and fled.

(Without-mitigating-circumstances condition)

The jury trial of a 52-year-old man who murdered his 82-year-old mother was held at the Osaka District Court. According to the prosecution, the man was suspected of strangling his mother with a rope and leaving her body on the banks of the Yodo River around 23:00 on May 22, 2022. Four years before the incident, the man was laid off and began living with his mother, who relied on a pension. As his mother’s life became increasingly difficult, she verbally abused him, insisting, "If you stay at home, you should earn money.” Despite the man’s explanations about his struggles to find a job, he felt mentally driven into a corner and decided to kill his mother out of sheer desperation. In the evening of the incident, he took his mother out for a walk and headed to the crime scene by train. Seeing that no one was around, he took out a rope from his bag, strangled her from behind, and fled.
