# Supplementary material for: Judges versus artificial intelligence in juror decision-making in criminal trials: Evidence from two pre-registered experiments
Source: PLoS One. 2025 Jan 30;20(1):e0318486. doi: 10.1371/journal.pone.0318486 (PMC11781698; doi:10.1371/journal.pone.0318486)
Supplement: S3 Appendix — Error bars indicate standard errors. (DOCX) [file pone.0318486.s003.docx]

**Supporting information**

**S3 Jury judgments in Experiment 1 (full sample analysis).**
